# *HTRA1* rs11528744, *BCRA1* rs9928736, and *B*3*GLCT* rs4381465 are associated with age-related macular degeneration in a Chinese population

**DOI:** 10.3389/fgene.2022.997840

**Published:** 2022-09-29

**Authors:** Guo Huang, Huan Li, Shuang Lai, Jialing Xiao, Liang Wang, Huijuan Xu, Chuntao Lei, Jinglan Zhang, Man Yu, Ping Shuai, Yuping Liu, Yi Shi, Kaijie Wang, Bo Gong

**Affiliations:** ^1^ Human Disease Genes Key Laboratory of Sichuan Province and Institute of Laboratory Medicine, Sichuan Academy of Medical Sciences and Sichuan Provincial People’s Hospital, University of Electronic Science and Technology of China, Chengdu, Sichuan, China; ^2^ Department of Health Management, Sichuan Academy of Medical Sciences and Sichuan Provincial People’s Hospital, University of Electronic Science and Technology of China, Chengdu, Sichuan, China; ^3^ Research Unit for Blindness Prevention of Chinese Academy of Medical Sciences (2019RU026), Sichuan Academy of Medical Sciences and Sichuan Provincial People’s Hospital, University of Electronic Science and Technology of China, Chengdu, Sichuan, China; ^4^ Department of Ophthalmology, Sichuan Academy of Medical Sciences and Sichuan Provincial People’s Hospital, University of Electronic Science and Technology of China, Chengdu, Sichuan, China; ^5^ Beijing Tongren Eye Center, Beijing Tongren Hospital, Capital Medical University, Beijing Ophthalmology and Visual Sciences Key Lab, Beijing, China

**Keywords:** single nucleotide polymorphisms (SNPs), age-related macular degeneration (AMD), case-control study, *HTRA1*, *BCRA1*, *B3GLCT*

## Abstract

**Purpose:** Age-related macular degeneration (AMD) is a leading cause of vision loss. A Previous study based on the co-localization analysis of the genome-wide association study (GWAS) and eQTL genetic signals have reported that single nucleotide polymorphisms (SNPs), including rs760975, rs11528744, rs3761159, rs7212510, rs6965458, rs7559693, rs56108400, rs28495773, rs9928736, rs11777697, rs4381465 are associated with AMD in Americans. The aim of this study was to investigate the association of these SNPs in a Han Chinese population.

**Methods:** There were 576 patients with wet AMD and 572 healthy controls collected in this study. All SNPs were genotyped by flight mass spectrum. Hardy–Weinberg equilibrium was applied to evaluate allele distributions for both AMD and control groups. The genotype and allele frequencies were evaluated using the χ^2^ tests. Odds ratio (OR) and 95% confidence intervals (95% CI) were calculated for the risk of genotype and allele.

**Results:** Three of the 11 SNPs (rs11528744 in *HTRA1,* rs9928736 in *BCRA1* and rs4381465 in *B3GLCT*) were found to be significantly associated with AMD in the allelic model (corrected *p* = 0.001, OR = 1.391, 95%CI = 1.179–1.640 for rs11528744; corrected *p* = 0.004, OR = 0.695, 95%CI = 0.544–0.888 for rs9928736; corrected *p* = 0.002, OR = 0.614, 95%CI = 0.448–0.841 for rs4381465). There were no differences for the remaining eight SNPs between AMD cases and healthy controls.

**Conclusion:** Our results showed that *HTRA1* rs11528744, *BCRA1* rs9928736, and *B3GLCT* rs4381465 were associated with wet AMD, suggesting that *HTRA1, BCRA1*, and *B3GLCT* genes may be involved in the development of AMD.

## Introduction

Age-related macular degeneration (AMD) is a progressive blinding disease and the leading cause of vision loss among the old people, influencing about 5% of those more than 75-year-old (W Smith et al., 2001). With population aging worldwide, it is estimated that the number of AMD patients will be increased to 196 million in 2020, 288 million in 2040. Since Asia accounts for more than half of the world’s population, the number of cases is expected to reach 113 million by 2040 (Wong et al., 2014). The decrease of the Retinal pigment epithelium (RPE) functions is a significant character of this disease and drusen are the hallmark lesions of the AMD each stage (Davis et al., 2005; Ferris et al., 2005). According to the size and quantity of drusen, the AMD can be divided into early, intermediate and advanced stages accompanied by the presence and absence of choroidal neovascularization. The advanced stage is divided into two categories: dry AMD and wet AMD. The term “dry AMD” refers to geographic atrophy (GA) change without neovascularization, while the wet AMD refers to the neovascular or exudative stage of this disease (Age-Related Eye Disease Study Research Group, 2001).

AMD is a multigenetic disease, identification of associated variants of this disease can contribute to uncover the pathogenesis and guide treatment. With advances in sequencing technology, more and more associated gene variants were discovered and verified (Arakawa et al., 2011; Raychaudhuri et al., 2011; Fritsche et al., 2013; Helgason et al., 2013; Seddon et al., 2013; van de Ven et al., 2013; Zhan et al., 2013). So far, at least 103 AMD associated loci have been identified according to genome-wide association study (GWAS) Catalog and other publications, with *CFH* and *ARMS2/HTRA1* to be the two most notable risk loci among these 103 identified AMD associated loci (Deng et al., 2022). A pervious GWAS identified 52 independently associated common and rare variants distributed across 34 loci that account for 46% of the genetic variance (Fritsche et al., 2016). GWAS loci are a valuable resource for understanding disease mechanisms. However, they can’t necessarily pinpoint the causal genes responsible for the disease association. EQTL can be used in conjunction with GWAS to facilitate the identification of candidate genes. A recent study of eQTL in macula-specific retina and RPE/choroid and single-nucleus RNA-seq from human retina and RPE sample identified 15 putative causal genes for 13 AMD risk loci, including the genes TSPAN10 and TRPM1 (Orozco et al., 2020). We found that among the 13 loci, the other 11 SNPs were not reported to be associated with AMD in Chinese population.

Therefore, in this study we aimed to investigate the possible involvement of these 11 selected SNPs with the risk of developing AMD in a Han Chinese population.

## Materials and methods

### Study subjects

We have recruited 576 patients with wet AMD and 572 healthy controls from the Sichuan Provincial People’s Hospital. Briefly, all the AMD patients received complete ophthalmic investigations and diagnosis of disease pathology was based on clinical findings of fluorescein angiographic and indocyanine green angiography. Inclusion criteria of the AMD patients were 1) women or men aged 55 older, 2) no association with other retinal disease (e.g., high myopia, diabetic retinopathy or macular dystrophies). Scoring criteria of AMD patients was carried out as per the AREDS criteria (Age-Related Eye Disease Studies). The control subjects who recruited from the Physical Examination Center were≧60 years old. They all received ophthalmic examination and were free from any eye diseases. All subjects recruited are Han Chinese living in Sichuan province in the southwest China. This study was approved by the Institutional Review Board from Sichuan Provincial People’s Hospital and informed consent was obtained from each individual prior to participation in this study. All procedures were carried out in accordance with the tenets of the Declaration of Helsinki.

### Single nucleotide polymorphisms selection

A previous study based on the co-localization analysis of GWAS and eQTL genetic signals have identified 15 putative causal genes for 13 AMD risk loci (Orozco et al., 2020). Among the 13 loci, 11 SNPs including rs760975, rs11528744, rs3761159, rs7212510, rs6965458, rs7559693, rs56108400, rs28495773, rs9928736, rs11777697, rs4381465 were not reported to be associated with AMD in Chinese population. In this study, we thus chose these 11 SNPs and evaluated the association with AMD in a Han Chinese population.

### Single nucleotide polymorphisms genotyping

Genomic DNA was extracted from blood which was drown from each subject with ethylenediaminetetraacetic acid (EDTA) vacuum blood collection tube by serial phenol/chloroform extraction and ethanol precipitation. The SNPs genotyping was performed with time-of-flight mass spectrometry (Mass array MALDI-TOF System, SEQUENOM, Inc.). Briefly, after multiplex PCR amplification, SNP sequence-specific primers were added to the products, and one base was extended at SNP site, and different genotypes extended different bases. Then, in a non-electric drift region, excited by an instantaneous nanosecond (10^−9^s) laser, the genes were separated according to their mass-to-charge ratio, and different genotypes were distinguished according to the different masses of the extended bases flying to the detector in the vacuum tube.

### Statistical analysis

All the statistical data were analyzed using SPSS 20.0 statistics software (IBM, Somers, NY). The Hardy–Weinberg equilibrium (HWE) and allelic or genotypic frequencies between cases and controls were tested by the Chi-square test. All differences are shown as mean difference ±standard deviation (SD). The Bonferroni method was conducted to perform correction for multiple comparisons whereby the *p* value was multiplied with the number of comparisons (corrected P). It was considered to be significant when corrected *p* was less than 0.05.

## Results

### Clinical data

**FIGURE 1:**
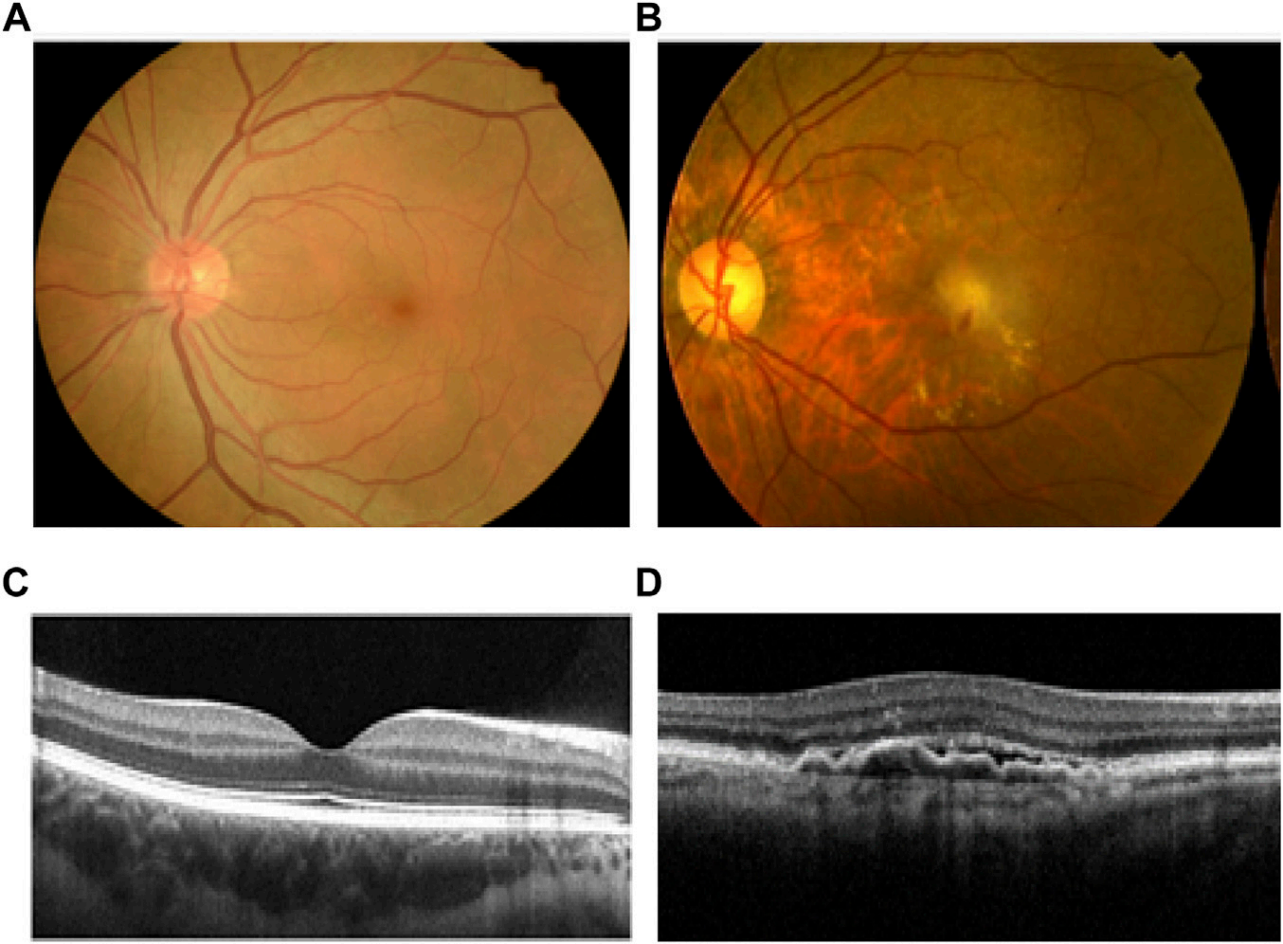
Comparison of health and AMD human retina. **(A)** Normal human fundus image of the left eye. **(B)** Fundus image of a patient with left eye wet AMD, with drusen, exudate, subretinal hemorrhage and scarring at the macular region. **(C)** OCT image of normal macula. **(D)** OCT image of wet AMD macula. AMD, age-related macular degeneration; OCT, optical coherence tomography.

A total of 576 AMD patients and 572 healthy controls were recruited in this study. A patient with AMD had drusen, exudate, subretinal hemorrhage and scarring at the macular region compared to that from the normal subject (Figure 1). The average age of healthy controls and AMD patients was 70.57 ± 9.58 (60.99–80.15) and 66.18 ± 9.52 (56.66–75.70) years old, respectively(*p* < 0.05). The percentage of female patients from AMD subjects was 33.16% while the proportion of female was 52.10% in the control group (*p* < 0.01). The BMI (Body Mass Index) of healthy controls and AMD patients was 22.91 ± 2.58 kg/m^2^ and 22.80 ± 2.36 kg/m^2^ (*p* > 0.05,Table 1).

**TABLE 1:** Basic Information of the groups.

Groups		Healthy controls	AMD cases	*p* Value
Age(year, mean ± SD)		70.57 ± 9.58	64.79±8.85	*p* < 0.05
gender	Male	275	398	*p* < 0.01
	Female	297	178	
BMI (kg/m2)		22.91 ± 2.58	22.80 ± 2.36	*p* > 0.05

### Single nucleotide polymorphisms analysis

Allele distributions of the 10 SNPs were in accordance with the HWE (*p* > 0.05) exception for rs6965458, which indicated that genotypes of these 10 SNPs fit the general population distribution and the samples were randomly selected within this region. The case-control analysis showed that three of the 11 selected SNPs had significant differences in distribution of allele frequency (*p* = 9 × 10^–5^, OR = 1.391, 95%CI = 1.179–1.640 for rs1152874; *p* = 0.004, OR = 0.695, 95%CI = 0.544–0.888 for rs9928736; *p* = 0.002, OR = 0.598, 95%CI = 0.430–0.725 from rs4381465) between AMD cases and healthy controls. After adjusting for multiple testing using Bonferroni correction, these three SNPs still showed significant associations between these two groups (Corrected *p* < 0.05, Tables 2, 3). For rs1152874(C), the minor allele frequency (MAF) was higher in AMD cases (0.500) than that in healthy controls (0.418), indicating that the minor allele (C) may be a risk factor for AMD. For rs9928736(C), the MAF was lower in AMD cases (0.112) than that in control group (0.153), for rs4381465 (A), the MAF was lower in AMD cases (0.060) than that in control group (0.094) as well, demonstrating that the minor allele of these two SNPs was a protective effect for AMD. We also conducted the statistical analyses to compare the sex (male or female) and age between AMD cases and healthy controls for the 11 selected SNPs. However, there were no significant differences for these three SNPs when compared the sex (male or female) and age between AMD cases and healthy controls (Corrected *p* > 0.05, Supplementary Tables S1–S6).

**TABLE 2:** Basic information and allele frequencies of the 11 selected SNPs.

SNP (gene name)	Chromosome	Gene locus	Allele	Minor allele frequency	
				Case	Control
rs760975 (BAIAP2L2)	22q13.1	38,071,777	G/C	0.181	0.174
rs11528744 (HTRA1)	10q21.2	122,534,138	C/T	0.500	0.418
rs3761159 (SLC12A5-AS1)	20q11.1	46,006,318	C/T	0.220	0.230
rs7212510 (TMEM199)	17q21.1	28,376,663	T/A	0.277	0.289
rs6965458 (PILRB)	7q11.23	100,375,779	A/G	0.030	0.039
rs7559693 (COL4A3)	2q14.1	227,254,233	G/C	0.291	0.247
rs56108400 (AC009779.3)	12q14	55,819,513	T/G	0.343	0.306
rs28495773 (PILRB)	7q11.23	100,345,960	T/A	0.027	0.035
rs9928736 (BCAR1)	16q11.2	75,208,831	C/T	0.112	0.153
rs11777697 (TNFRSF10A)	8q13	23,231,471	G/C	0.354	0.318
rs4381465 (B3GLCT)	13q21.1	31,133,338	A/T	0.060	0.094

The SNPs, positions were from the Human Assembly February 2009 (GRCh37/hg19).

**TABLE 3:** The genotype frequencies and association analysis of 11 SNPs in AMD cases and controls.

SNP (gene name)	Genotype frequency (%)			HWE		Allele	Corrected	Or (95%CI)
	Genotype	AMD cases	Healthy controls	AMD	Controls	P	P	
rs760975 (BAIAP2L2)	GG	28(0.049)	21(0.037)	0.009	0.275	0.695	7.645	1.044
	GC	152(0.274)	156(0.273)					(0.842–1.294)
	CC	396(0.677)	391(0.684)					
rs11528744 (HTRA1)	CC	151(0.267)	104(0.182)	0.243	0.443	0	0	1.391
	CT	274(0.444)	268(0.469)					(1.179–1.640)
	TT	151(0.288)	197(0.344)					
rs3761159 (SLC12A5-AS1)	CC	24(0.045)	30(0.052)	0.346	0.97	0.573	6.303	0.945
	CT	205(0.351)	202(0.353)					(0.777–1.150)
	TT	345(0.601)	337(0.589)					
rs7212510 (TMEM199)	TT	47(0.080)	45(0.079)	0.527	0.602	0.504	5.544	0.940
	TA	224(0.375)	239(0.418)					(0.783–1.127)
	AA	304(0.542)	285(0.498)					
rs6965458 (PILRB)	AA	2(0.003)	3(0.005)	0.038	0.015	0.277	3.047	0.779
	AG	31(0.056)	38(0.066)					(0.496–1.224)
	GA	542(0.941)	527(0.921)					
rs7559693 (COL4A3)	GG	44(0.073)	27(0.047)	0.353	0.081	0.019	0.209	1.248
	GC	246(0.441)	227(0.397)					(1.037–1.503)
	CC	284(0.483)	314(0.549)					
rs56108400 (AC009779.3)	TT	65(0.101)	48(0.084)	0.615	0.304	0.058	0.638	1.185
	TG	265(0.458)	252(0.441)					(0.994–1.411)
	GG	246(0.441)	269(0.470)					
rs28495773 (PILRB)	TT	1(0)	1(0.002)	0.358	0.68	0.306	3.366	0.779
	TA	29(0.052)	37(0.065)					(0.483–1.258)
	AA	542(0.941)	527(0.921)					
rs9928736 (BCAR1)	CC	10(0.010)	15(0.026)	0.218	0.569	0.004	0.044	0.695
	CT	107(0.188)	143(0.250)					(0.544–0.888)
	TT	452(0.788)	407(0.712)					
rs11777697 (TNFRSF10A)	GG	55(0.104)	62(0.108)	0.002	0.393	0.07	0.77	1.174
	GC	297(0.507)	238(0.416)					(0.987–1.397)
	CC	223(0.385)	269(0.470)					
rs4381465 (B3GLCT)	AA	1(0.003)	3(0.005)	0.429	0.316	0.002	0.022	0.614
	AT	67(0.125)	101(0.177)					(0.448–0.841)
	TT	507(0.868)	464(0.811)					

HWE, Hardy–Weinberg equilibrium; *p* < 0.05 was considered significant, Bonferroni multiple comparison.

Four different genetic models (homozygous, heterozygous, dominant and recessive models) were used to further explore the relationship between these three SNPs and AMD (Table 4). After Bonferroni correction, we found that the homozygous model (CC/TT, *p* = 1 × 10^–6^, OR = 1.894, 95% CI = 1.365–2.628), dominant model (CC + CT/TT, *p* = 0.002, OR = 1.491, 95% CI = 1.567–1.807) and recessive (CC/CT + TT, *p* = 0.001, OR = 1.589, 95% CI = 1.198–1.926) model for rs1152874 showed high risk in the AMD, indicating that subjects carrying rs11528744 CC/TT or CC/CT genotypes were more likely to be suffered from AMD. Significant association of rs9928736 was detected in the heterozygote model (CT/TT, *p* = 0.006, OR = 0.674, 95% CI = 0.507–0.895) and dominant models (CC + CT/TT, *p* = 0.004, OR = 0.667, 95% CI = 0.507–0.808) for rs9928736 between AMD cases and healthy controls, showing that subjects carrying CT and CC + CT genotypes may be susceptible to the disease than those carrying TT genotypes for rs9928736. The significant difference for rs4381465 was found in heterozygote model (AT/TT, *p* = 0.003, OR = 0.607, 95% CI = 0.435–0.847) and dominant model (AA + AT/TT, *p* = 0.002, OR = 0.598, 95% CI = 0.430–0.725) between AMD cases and healthy controls, suggesting that subjects carrying AT genotypes and AA + AT were more likely to suffer from PDR than those carrying TT genotypes for rs4381465.

**TABLE 4:** Association analysis between three SNPs and AMD in four genetic models.

SNP (gene name)	Homozygote		Heterozygote		Dominant		Recessive	
	P	OR(95%CI)	P	OR(95%CI)	P	OR(95%CI)	P	OR(95%CI)
rs11528744(C)	0.000	1.894(1.365–2.628)	0.037	1.334(1.017–1.749)	0.002	1.491(1.567–1.807)	0.001	1.589(1.198–1.926)
(HTRA1)								
rs9928736(C)	0.213	0.600(0.267–1.351)	0.006	0.674(0.507–0.895)	0.004	0.667(0.507–0.808)	0.303	0.656(0.292–0.795)
(BCAR1)								
rs4381465(A)	0.277	0.305(0.0.32–0.943)	0.003	0.607(0.435–0.847)	0.002	0.598(0.430–0.725)	0.311	0.328(0.034–0.398)
(B3GLCT)								

OR, odds ratio; CI, confidence interval; A, minor allele; B, major allele. Genotype (AA/AB/BB) analyses were conducted for the homozygous model (AA, compared with BB), heterozygous model (AB, compared with BB), dominant model (AA + AB, compared with BB), and recessive model (AA, compared with AB + BB). *p* < 0.0125 was considered significant, Bonferroni multiple comparisons.

## Discussion

A Previous study based on the co-localization analysis of the GWAS and eQTL genetic signals have identified 15 putative causal genes for 13 AMD risk loci (Orozco et al., 2020). In present study, we utilized flight mass spectrometry to compare the frequencies of 11 SNPs among these 13 AMD risk loci between 576 AMD patients and 572 healthy controls from a Chinese Han population. This case–control study showed that obvious differences for *HTRA1* rs11528744 were found in the allelic distributions and under the dominant, homozygote and recessive model between AMD cases and healthy controls. In addition, obvious differences for *BCRA1* rs9928736 and *B3GLCT* rs4381465 were found in the allelic distributions and under the heterozygous and dominant model between AMD cases and healthy controls. These results suggested that the genetic susceptibility to AMD may be due to the presence of the rs11528744 variants of the *HTRA1* gene, the rs9928736 variants of the *BCRA1* gene and the rs4381465 variant of the *B3GLCT* gene.

The SNP rs11528744 was significantly associated with AMD Chinese cohort in this study. The presence of the C allele in rs11528744 was associated with a higher risk of AMD cases. The frequency of the rs11528744 minor C-allele is 0.741 in the population of Africa, 0.614 in the Europe and 0.499 in the Asian, respectively (www.ncbi.nlm.nih.gov/snp/rs11528744#frequency_tab). According to the database comprising eQTL from both human RPE/choroid and retina, the SNP rs11528744 was significantly associated with *HTRA1* genes (*p* = 0.000, MAF = 0.37, Beta = 0.3) (http://eye-eqtl.com/). The high-temperature requirement factor A(*HTRA1*), which encodes a heat shock serine protease that is activated by cellular stress and is expressed in the mouse and human retina (Yoshida et al., 2002; Yoshida and Sasakawa, 2003). Previous research suggested that HTRA1 plays a role in AMD pathogenesis by regulating the TGF-β pathway. TGF-β is considered involving in cell proliferation, angiogenesis and extracellular matrix deposition (Friedrich et al., 2015). What’s more, the binding and inhibition of TGF-β may result in the overexpression of *HTRA1* gene in wet AMD (Xu et al., 2008). Possibly, the increased expression levels of HTRA1 mRNA and protein may influence the integrity of Bruch’s membrane, promoting the advancement of CNV stage (Dewan et al., 2006). Together, these findings support a key role for HTRA1 in AMD susceptibility and identify a potential marker for AMD pathogenesis.

The SNP rs9928736 was also significantly associated with AMD Chinese cohort in present study. The presence of the C allele in rs9928736 was associated with a protective effect of AMD cases. The frequency of the rs9928736 minor C-allele is 0.250 in the European population, 0.378 in the African and 0.114 in the Asian, respectively (www.ncbi.nlm.nih.gov/snp/rs9928736#frequency_tab). According to the database comprising eQTL from both human macula and retina, the SNP rs9928736 was significantly associated with *BCRA1* genes (*p* = 0.000, MAF = 0.25, Beta = 0.36) (http://eye-eqtl.com/). One of the CAS protein family members, breast cancer anti-estrogen resistance 1 (BCAR1), was identified as a 130 kDa cellular protein and to be hyperphosphorylated in v-Crk and v-Src transformed cells (Reynolds et al., 1989; Kanner et al., 1991). Initially, various studies about BCAR1 was mostly focus on the association with breast cancer and lung cancer. Recently, *BCAR1* was discovered to be expressed in the retina and to be important in early retinal development, and a Bcar1 mouse model showed significant disruption of the ganglion cell laye (Riccomagno et al., 2014). What’s else, a research reported that a non-synonymous and splice variant was found in *BCAR1* in two Michigan families by the whole exome sequencing and this rare variant in *BCAR1* is also remarkable as the most likely candidate at the AMD-GWAS loci (Ratnapriya et al., 2020).

Another SNP suggestively associated with AMD in our study is rs4381465. The SNP rs4381465 was significantly associated with AMD Chinese cohort in this study. The presence of the A allele in rs4381465 was associated with a protective effect of AMD cases. The frequency of the rs4381465 minor A-allele is 0.409 in the European population, 0.143 in the African and 0.01 in the Asian, respectively (www.ncbi.nlm.nih.gov/snp/rs4381465#frequency_tab). According to the database comprising eQTL from both human macula and retina, the SNP rs4381465 was significantly associated with *B3GLCT* genes (*p* = 0.000, MAF = 0.43, Beta = −0.53) (http://eye-eqtl.com/). one to three glycosyltransferase (B3GLCT) can collaborate with POFUT2 in the endoplasmic reticulum to add an O-linked glucose-1-3fucose disaccharide at serine or threonine residues to appropriately folded Thrombospondin Type-1 Repeat (TSR) domains. (Lauwen et al., 2021; Neupane et al., 2021). A previous GWAS reported that the SNP 9564692 is located in the *B3GLCT* gene and the MAF of rs9564692 is associated with a decreased risk for AMD (OR = 0.89, *p* = 3.3 × 10^–10^) (Fritsche et al., 2016). Furthermore, recent transcriptome-wide analysis combined with eQTL mapping revealed that the MAF of rs9564692 is linked to lower levels of *B3GLCT* RNA expression in the retina (Ratnapriya et al., 2019). Taken together, these data show that lower These data show that lower B3GLCT levels are protective against AMD. levels are protective for AMD and *B3GLCT* gene may be involved in the pathogenesis of AMD.

Our study also had several limitations. We only chose SNPs that have been previously reported and no new SNPs were found. Furthermore, no functional work of these 3 -SNP loci were done. In the future, we need attempts to understand the role of these SNPs in the development of AMD in Han population. Despite all of our reported genetic association of the SNPs with the outcome, it must be admitted that most of the analyses were underpowered which may be one explanation why the other eight SNPs did not replicate the association observed in previous studies.

In conclusion, We found that the polymorphisms of *HTRA1* rs11528744, *BCRA1* rs9928736, and B*3GLCT* rs4381465 were associated with AMD significantly in a Han Chinese population. Future study including more functional experiments is needed to elucidate these genes involved in the pathogenesis of AMD.

## Data Availability

Original datasets are available in a publicly accessible repository. The original contributions presented in the study are publicly available. This data can be found here: https://www.ncbi.nlm.nih.gov/bioproject/?term=+PRJNA882186.

## References

[B1] Age-Related Eye Disease Study Research Group (2001). The age-related eye disease study (AREDS) System for classifying cataracts from photographs: AREDS report No. 4. Am. J. Ophthalmol. 131 (2), 167–175. 10.1016/s0002-9394(00)00732-7 11228291 PMC2032014

[B2] Arakawa S. Takahashi A. Ashikawa K. Hosono N. Aoi T. Yasuda M. (2011). Genome-wide association study identifies two susceptibility loci for exudative age-related macular degeneration in the Japanese population. Nat. Genet. 43, 1001–1004. 10.1038/ng.938 21909106

[B3] Davis M. D. Gangnon R. E. Lee L-Y. Hubbard L. D. Klein B. E. K. Klein R. (2005). The age-related eye disease study severity scale for age-related macular degeneration: AREDS report No. 17. Arch. Ophthalmol. 123 (11), 1484–1498. 10.1001/archopht.123.11.1484 16286610 PMC1472813

[B4] Deng Y. Qiao L. Du M. Qu C. Wan L. Li J. (2022). Age-related macular degeneration: Epidemiology, genetics, pathophysiology, diagnosis, and targeted therapy. Genes Dis. 9, 62–79. 10.1016/j.gendis.2021.02.009 35005108 PMC8720701

[B5] Dewan A. Liu M. Hartman S. Zhang S. S. Liu D. T. Zhao C. (2006). HTRA1 promoter polymorphism in wet age-related macular degeneration. Science 314, 989–992. 10.1126/science.1133807 17053108

[B6] Ferris F. L. Davis M. D. Clemons T. E. Lee L-Y. Chew E. Y. Lindblad A. S. (2005). A simplified severity scale for age-related macular degeneration: AREDS report No. 18. Arch. Ophthalmol. 123 (11), 1570–1574. 10.1001/archopht.123.11.1570 16286620 PMC1473206

[B7] Friedrich U. Datta S. Schubert T. Plossl K. Schneider M. Grassmann F. (2015). Synonymous variants in HTRA1 implicated in AMD susceptibility impair its capacity to regulate TGF-beta signaling. Hum. Mol. Genet. 24, 6361–6373. 10.1093/hmg/ddv346 26310622

[B8] Fritsche L. G. Chen W. Schu M. Yaspan B. L. Yu Y. Thorleifsson G. (2013). Seven new loci associated with age-related macular degeneration. Nat. Genet. 45, 433–439. 10.1038/ng.2578 23455636 PMC3739472

[B9] Fritsche L. G. Igl W. Bailey J. N. Grassmann F. Sengupta S. Bragg-Gresham J. L. (2016). A large genome-wide association study of age-related macular degeneration highlights contributions of rare and common variants. Nat. Genet. 48, 134–143. 10.1038/ng.3448 26691988 PMC4745342

[B10] Helgason H. Sulem P. Duvvari M. R. Luo H. Thorleifsson G. Stefansson H. (2013). A rare nonsynonymous sequence variant in C3 is associated with high risk of age-related macular degeneration. Nat. Genet. 45, 1371–1374. 10.1038/ng.2740 24036950

[B11] Kanner S. B. Reynolds A. B. Parsons J. T. (1991). Tyrosine phosphorylation of a 120-kilodalton pp60src substrate upon epidermal growth factor and platelet-derived growth factor receptor stimulation and in polyomavirus middle-T-antigen-transformed cells. Mol. Cell. Biol. 11, 713–720. 10.1128/mcb.11.2.713 1703631 PMC359722

[B12] Lauwen S. Baerenfaenger M. Ruigrok S. de Jong E. K. Wessels H. den Hollander A. I. (2021). Loss of the AMD-associated B3GLCT gene affects glycosylation of TSP1 without impairing secretion in retinal pigment epithelial cells. Exp. Eye Res. 213, 108798. 10.1016/j.exer.2021.108798 34695439

[B13] Neupane S. Goto J. Berardinelli S. J. Ito A. Haltiwanger R. S. Holdener B. C. (2021). Hydrocephalus in mouse B3glct mutants is likely caused by defects in multiple B3GLCT substrates in ependymal cells and subcommissural organ. Glycobiology 31, 988–1004. 10.1093/glycob/cwab033 33909046 PMC8579228

[B14] Orozco L. D. Chen H. H. Cox C. Katschke K. J. Jr. Arceo R. Espiritu C. (2020). Integration of eQTL and a single-cell atlas in the human eye identifies causal genes for age-related macular degeneration. Cell. Rep. 30, 1246–1259. 10.1016/j.celrep.2019.12.082 31995762

[B15] Ratnapriya R. Acar I. E. Geerlings M. J. Branham K. Kwong A. Saksens N. T. M. (2020). Family-based exome sequencing identifies rare coding variants in age-related macular degeneration. Hum. Mol. Genet. 29, 2022–2034. 10.1093/hmg/ddaa057 32246154 PMC7390936

[B16] Ratnapriya R. Sosina O. A. Starostik M. R. Kwicklis M. Kapphahn R. J. Fritsche L. G. (2019). Retinal transcriptome and eQTL analyses identify genes associated with age-related macular degeneration. Nat. Genet. 51, 606–610. 10.1038/s41588-019-0351-9 30742112 PMC6441365

[B17] Raychaudhuri S. Iartchouk O. Chin K. Tan P. L. Tai A. K. Ripke S. (2011). A rare penetrant mutation in CFH confers high risk of age-related macular degeneration. Nat. Genet. 43, 1232–1236. 10.1038/ng.976 22019782 PMC3225644

[B18] Reynolds A. B. Roesel D. J. Kanner S. B. Parsons J. T. (1989). Transformation-specific tyrosine phosphorylation of a novel cellular protein in chicken cells expressing oncogenic variants of the avian cellular src gene. Mol. Cell. Biol. 9, 629–638. 10.1128/mcb.9.2.629 2469003 PMC362640

[B19] Riccomagno M. M. Sun L. O. Brady C. M. Alexandropoulos K. Seo S. Kurokawa M. (2014). Cas adaptor proteins organize the retinal ganglion cell layer downstream of integrin signaling. Neuron 81, 779–786. 10.1016/j.neuron.2014.01.036 24559672 PMC3988023

[B20] Seddon J. M. Yu Y. Miller E. C. Reynolds R. Tan P. L. Gowrisankar S. (2013). Rare variants in CFI, C3 and C9 are associated with high risk of advanced age-related macular degeneration. Nat. Genet. 45, 1366–1370. 10.1038/ng.2741 24036952 PMC3902040

[B21] van de Ven J. P. Nilsson S. C. Tan P. L. Buitendijk G. H. Ristau T. Mohlin F. C. (2013). A functional variant in the CFI gene confers a high risk of age-related macular degeneration. Nat. Genet. 45, 813–817. 10.1038/ng.2640 23685748

[B22] W Smith J. A. Klein R. Mitchell P. Klaver C. C. Klein B. E. Hofman A. (2001). Risk factors for age-related macular degeneration: Pooled findings from three continents. Ophthalmology 108 (4), 697–704. 10.1016/s0161-6420(00)00580-7 11297486

[B23] Wong W. L. Su X. Li X. Cheung C. M. G. Klein R. Cheng C.-Y. (2014). Global prevalence of age-related macular degeneration and disease burden projection for 2020 and 2040: A systematic review and meta-analysis. Lancet. Glob. Health 2, e106–e116. 10.1016/S2214-109X(13)70145-1 25104651

[B24] Xu Y. Guan N. Xu J. Yang X. Ma K. Zhou H. (2008). Association of CFH, LOC387715, and HTRA1 polymorphisms with exudative age-related macular degeneration in a northern Chinese population. Mol. Vis. 14, 1373–1381. 18682812 PMC2493029

[B25] Yoshida S. Katayama E. Kuwae A. Mimuro H. Suzuki T. Sasakawa C. (2002). Shigella deliver an effector protein to trigger host microtubule destabilization, which promotes Rac1 activity and efficient bacterial internalization. EMBO J. 21, 2923–2935. 10.1093/emboj/cdf319 12065406 PMC126072

[B26] Yoshida S. Sasakawa C. (2003). Exploiting host microtubule dynamics: A new aspect of bacterial invasion. Trends Microbiol. 11, 139–143. 10.1016/s0966-842x(03)00023-4 12648946

[B27] Zhan X. Larson D. E. Wang C. Koboldt D. C. Sergeev Y. V. Fulton R. S. (2013). Identification of a rare coding variant in complement 3 associated with age-related macular degeneration. Nat. Genet. 45, 1375–1379. 10.1038/ng.2758 24036949 PMC3812337

