# Supplementary material for: HTRA1 rs11528744, BCRA1 rs9928736, and B3GLCT rs4381465 are associated with age-related macular degeneration in a Chinese population
Source: Front Genet. 2022 Sep 29;13:997840. doi: 10.3389/fgene.2022.997840 (PMC9574478; doi:10.3389/fgene.2022.997840)
Supplement: Supplementary file 1 [file DataSheet1.docx]

**Table S1 The genotype frequencies and association analysis of 11 SNPs in female AMD cases and controls**

| SNP  (gene name) | Genotype frequency (%) | | | HWE | | Allele P | Corrected | OR (95%CI) |
| --- | --- | --- | --- | --- | --- | --- | --- | --- |
|  | Genotype | Female Cases | Healthy Controls | AMD | Controls |  | P |  |
| rs760975  (BAIAP2L2) | GG | 12（0.063） | 11（0.037） | 0.005 | 0.261 | 0.605 | 6.655 | 1.093（0.779-1.533） |
|  | GC | 45（0.236） | 77（0.261） |  |  |  |  |  |
|  | CC | 134（0.702） | 207（0.702） |  |  |  |  |  |
| rs11528744  (HTRA1) | CC | 48（0.251） | 55（0.186） | 0.828 | 0.196 | 0.008 | 0 | 1.423（1.098-1.844） |
|  | CT | 94（0.492） | 132（0.447） |  |  |  |  |  |
|  | TT | 49（0.257） | 108 (0.366) |  |  |  |  |  |
| rs3761159  (SLC12A5-AS1) | CC | 9（0.047） | 18 (0.061) | 0.643 | 0.599 | 0.851 | 0.088 | 0.971（0.716-1.317） |
|  | CT | 70（0.366） | 103 (0.349) |  |  |  |  |  |
|  | TT | 112（0.586） | 174 (0.590) |  |  |  |  |  |
| rs7212510  (TMEM199) | TT | 23（0.120） | 24 (0.0781) | 0.006 | 0.889 | 0.855 | 9.405 | 0.974（0.732-1.295） |
|  | TA | 62（0.325） | 122 (0.414) |  |  |  |  |  |
|  | AA | 106（0.555） | 149 (0.505) |  |  |  |  |  |
| rs6965458  (PILRB) | AA | 0（0.000） | 3（0.010） | 0.710 | 0.001 | 0.149 | 1.639 | 0.583（0.278-1.243） |
|  | AG | 10（0.052） | 20（0.068） |  |  |  |  |  |
|  | GG | 181（0.948） | 272 (0.922) |  |  |  |  |  |
| rs7559693  (COL4A3) | GG | 17 (0.089) | 14 (0.047) | 0.915 | 0.138 | 0.051 | 0.561 | 1.275（0.957-1.698） |
|  | GC | 81 (0.424) | 121 (0.410) |  |  |  |  |  |
|  | CC | 93 (0.487) | 160 (0.542) |  |  |  |  |  |
| rs56108400  (AC009779.3) | TT | 20 (0.105) | 29 (0.098) | 0.315 | 0.497 | 0.116 | 1.276 | 1.246（0.947-1.640） |
|  | TG | 93 (0.487) | 119 (0.403) |  |  |  |  |  |
|  | GG | 78 (0.408) | 147 (0.498) |  |  |  |  |  |
| rs28495773  (PILRB) | TT | 0 (0.000) | 1 (0.003) | 0.710 | 0.445 | 0.230 | 2.530 | 0.634（0.300-1.341） |
|  | TA | 10 (0.052) | 22 (0.075) |  |  |  |  |  |
|  | AA | 181(0.948) | 272(0.922) |  |  |  |  |  |
| rs9928736  (BCAR1) | CC | 2（0.010） | 8 (0.027) | 0.929 | 0.418 | 0.034 | 0.374 | 0.647（0.432-0.971） |
|  | CT | 34（0.178） | 70 (0.237) |  |  |  |  |  |
|  | TT | 155（0.812） | 217 (0.736) |  |  |  |  |  |
| rs11777697  (TNFRSF10A) | GG | 16（0.084） | 34 (0.115) | 0.017 | 0.579 | 0.479 | 5.269 | 1.103（0.841-1.447） |
|  | GC | 102（0.534） | 126 (0.427) |  |  |  |  |  |
|  | CC | 73（0.382） | 135 (0.458) |  |  |  |  |  |
| rs4381465  (B3GLCT) | AA | 0 (0.000) | 2 (0.007) | 0.354 | 0.469 | 0.029 | 0.319 | 0.581（0.356-0.950） |
|  | AT | 24 (0.126) | 57 (0.193) |  |  |  |  |  |
|  | TT | 167 (0.874) | 236 (0.800) |  |  |  |  |  |

**Table S2 The genotype frequencies and association analysis of 11 SNPs in male AMD cases and controls**

| SNP  (gene name) | Genotype frequency (%) | | | HWE | | Allele P | Corrected | OR (95%CI) |
| --- | --- | --- | --- | --- | --- | --- | --- | --- |
|  | Genotype | Male Cases | Healthy Controls | AMD | Controls |  | P |  |
| rs760975  (BAIAP2L2) | GG | 16（0.042） | 10（0.037） | 0.217 | 0.686 | 0.932 | 10.252 | 0.988（0.742-1.314） |
|  | CG | 106（0.277） | 79（0.290） |  |  |  |  |  |
|  | CC | 261（0.681） | 183（0.673） |  |  |  |  |  |
| rs11528744  (HTRA1) | CC | 101（0.264） | 49（0.180） | 0.202 | 0.907 | 0.011 | 0.121 | 1.331（1.067-1.660） |
|  | CT | 179（0.467） | 134（0.493） |  |  |  |  |  |
|  | TT | 103（0.269） | 89 (0.327) |  |  |  |  |  |
| rs3761159  (SLC12A5-AS1) | CC | 15（0.039） | 12 (0.044) | 0.402 | 0.558 | 0.702 | 7.722 | 0.950（0.729-1.238） |
|  | CT | 135（0.352） | 98 (0.360) |  |  |  |  |  |
|  | TT | 233（0.608） | 162 (0.596) |  |  |  |  |  |
| rs7212510  (TMEM199) | TT | 23（0.060） | 21 (0.077) | 0.156 | 0.567 | 0.485 | 5.335 | 0.917（0.718-1.170） |
|  | TA | 163（0.426） | 116 (0.426) |  |  |  |  |  |
|  | AA | 197（0.514） | 135 (0.496) |  |  |  |  |  |
| rs6965458  (PILRB) | AA | 2（0.005） | 1（0.004） | 0.010 | 0.279 | 0.686 | 7.546 | 0.884（0.486-1.609） |
|  | AG | 21（0.055） | 18（0.066） |  |  |  |  |  |
|  | GG | 360（0.940） | 253 (0.930) |  |  |  |  |  |
| rs7559693  (COL4A3) | GG | 25 (0.066) | 14 (0.051) | 0.210 | 0.459 | 0.141 | 1.551 | 1.208（0.939-1.553） |
|  | GC | 163 (0.430) | 105 (0.386) |  |  |  |  |  |
|  | CC | 191 (0.504) | 153 (0.563) |  |  |  |  |  |
| rs56108400  (AC009779.3) | TT | 43 (0.113) | 20 (0.074) | 0.877 | 0.044 | 0.396 | 4.356 | 1.107（0.875-1.400） |
|  | TG | 172 (0.451) | 132 (0.485) |  |  |  |  |  |
|  | GG | 166 (0.436) | 120 (0.441) |  |  |  |  |  |
| rs28495773  (PILRB) | TT | 2 (0.000) | 0 (0.000) | 0.004 | 0.572 | 0.767 | 8.437 | 0.909（0.486-1.702） |
|  | TA | 19 (0.050) | 18 (0.066) |  |  |  |  |  |
|  | AA | 360 (0.945) | 254 (0.934) |  |  |  |  |  |
| rs9928736  (BCAR1) | CC | 10（0.026） | 9 (0.033) | 0.039 | 0.598 | 0.016 | 0.176 | 0.683（0.500-0.933） |
|  | CT | 73（0.192） | 74 (0.272) |  |  |  |  |  |
|  | TT | 298（0.782） | 189 (0.695) |  |  |  |  |  |
| rs11777697  (TNFRSF10A) | GG | 40（0.104） | 29 (0.107) | 0.045 | 0.436 | 0.076 | 0.836 | 1.236（0.978-1.562） |
|  | GC | 194（0.507） | 111 (0.408) |  |  |  |  |  |
|  | CC | 149（0.389） | 132 (0.485) |  |  |  |  |  |
| rs4381465  (B3GLCT) | AA | 2 (0.005) | 2 (0.007) | 0.575 | 0.981 | 0.067 | 0.737 | 0.676（0.442-01.031） |
|  | AT | 42 (0.110) | 43 (0.158) |  |  |  |  |  |
|  | TT | 339 (0.885) | 227 (0.835) |  |  |  |  |  |

**Table S3 The genotype frequencies and association analysis of 11 SNPs in AMD cases and controls（40-60 years）**

| SNP  (gene name) | Genotype frequency (%) | | | HWE | | Allele P | Corrected | OR (95%CI) |
| --- | --- | --- | --- | --- | --- | --- | --- | --- |
|  | Genotype | AMD cases | Healthy Controls | AMD | Controls |  | P |  |
| rs760975  (BAIAP2L2) | GG | 7（0.038） | 4 （0.041） | 0.367 | 0.838 | 0.648 | 7.128 | 0.534（0.546-1.335） |
|  | GC | 48（0.264） | 30（0.306） |  |  |  |  |  |
|  | CC | 127（0.698） | 64（0.653） |  |  |  |  |  |
| rs11528744  (HTRA1) | CC | 45（0.247） | 16（0.163） | 0.114 | 0.590 | 0.071 | 0.781 | 1.384（0.972-1.970） |
|  | CT | 80（0.440） | 44（0.449） |  |  |  |  |  |
|  | TT | 57（0.313） | 38 (0.388) |  |  |  |  |  |
| rs3761159  (SLC12A5-AS1) | CC | 12（0.066） | 4 (0.041) | 0.653 | 0.506 | 0.305 | 3.355 | 1.236（0.824-1.855） |
|  | CT | 74（0.407） | 37 (0.378) |  |  |  |  |  |
|  | TT | 96（0.527） | 57 (0.582) |  |  |  |  |  |
| rs7212510  (TMEM199) | TT | 19（0.104） | 8 (0.082) | 0.065 | 0.776 | 0.961 | 10.571 | 1.009（0.685-1.489） |
|  | TA | 63（0.346） | 38 (0.388) |  |  |  |  |  |
|  | AA | 100（0.549） | 52 (0.531) |  |  |  |  |  |
| rs6965458  (PILRB) | AA | 0（0.000） | 0（0.000） | 0.732 | 0.714 | 0.457 | 5.027 | 0.685（0.251-1.867） |
|  | AG | 9（0.049） | 7（0.071） |  |  |  |  |  |
|  | GG | 173（0.951） | 91 (0.929) |  |  |  |  |  |
| rs7559693  (COL4A3) | GG | 14 (0.077) | 4 (0.041) | 0.944 | 0.206 | 0.591 | 6.501 | 1.115（0.751-1.655） |
|  | GC | 72 (0.398) | 42 (0.429) |  |  |  |  |  |
|  | CC | 95 (0.525) | 52 (0.531) |  |  |  |  |  |
| rs56108400  (AC009779.3) | TT | 15 (0.082) | 6 (0.061) | 0.501 | 0.322 | 0.635 | 6.985 | 1.097（0.749-1.607） |
|  | TG | 81 (0.445) | 44 (0.449) |  |  |  |  |  |
|  | GG | 86 (0.473) | 48 (0.490) |  |  |  |  |  |
| rs28495773  (PILRB) | TT | 1 (0.005) | 1 (0.010) | 0.017 | 0.067 | 0.250 | 2.750 | 0.587（0.234-1.470） |
|  | TA | 8 (0.044) | 7 (0.071) |  |  |  |  |  |
|  | AA | 173 (0.951) | 90 (0.918) |  |  |  |  |  |
| rs9928736  (BCAR1) | CC | 4（0.093） | 4 (0.112) | 0.065 | 0.305 | 0.026 | 0.286 | 0.563（0.337-0.938） |
|  | CT | 28（0.538） | 24 (0.469) |  |  |  |  |  |
|  | TT | 150（0.368） | 70 (0.418) |  |  |  |  |  |
| rs11777697  (TNFRSF10A) | GG | 17（0.104） | 11 (0.108) | 0.026 | 0.723 | 0.711 | 7.821 | 1.071（0.745-1.3541） |
|  | GC | 98（0.507） | 46 (0.416) |  |  |  |  |  |
|  | CC | 67（0.385） | 41 (0.470) |  |  |  |  |  |
| rs4381465  (B3GLCT) | AA | 1 (0.005) | 1 (0.010) | 0.732 | 0.738 | 0.302 | 3.322 | 0.710（0.369-1.364） |
|  | AT | 21 (0.115) | 15 (0.153) |  |  |  |  |  |
|  | TT | 160 (0.879) | 82 (0.837) |  |  |  |  |  |

HWE: Hardy–Weinberg equilibrium; P < 0.05 was considered significant, Bonferroni multiple comparisons.

**Table S4 The genotype frequencies and association analysis of 11 SNPs in AMD cases and controls（61-70 years）**

| SNP  (gene name) | Genotype frequency (%) | | | HWE | | Allele P | Corrected | OR (95%CI) |
| --- | --- | --- | --- | --- | --- | --- | --- | --- |
|  | Genotype | AMD cases | Healthy Controls | AMD | Controls |  | P |  |
| rs760975  (BAIAP2L2) | GG | 13（0.074） | 3（0.020） | 0.002 | 0.369 | 0.487 | 5.357 | 1.151（0.774-1.712） |
|  | GC | 42（0.240） | 47（0.307） |  |  |  |  |  |
|  | CC | 175（0.686） | 103（0.673） |  |  |  |  |  |
| rs11528744  (HTRA1) | CC | 39（0.223） | 30（0.196） | 0.861 | 0.430 | 0.261 | 2.871 | 1.193（0.876-1.626） |
|  | CT | 86（0.491） | 70（0.458） |  |  |  |  |  |
|  | TT | 50（0.286） | 53 (0.346) |  |  |  |  |  |
| rs3761159  (SLC12A5-AS1) | CC | 3（0.017） | 10 (0.065) | 0.149 | 0.424 | 0.120 | 1.320 | 0.741（0.507-1.082） |
|  | CT | 58（0.331） | 51 (0.333) |  |  |  |  |  |
|  | TT | 114（0.651） | 92 (0.601) |  |  |  |  |  |
| rs7212510  (TMEM199) | TT | 12（0.069） | 12 (0.078) | 0.890 | 0.170 | 0.125 | 1.375 | 0.768（0.548-1.076） |
|  | TA | 69（0.394） | 74 (0.484) |  |  |  |  |  |
|  | AA | 94（0.537） | 67 (0.438) |  |  |  |  |  |
| rs6965458  (PILRB) | AA | 0（0.000） | 0（0.000） | 0.697 | 0.708 | 0.949 | 10.439 | 0.971（0.389-2.421） |
|  | AG | 10（0.057） | 9（0.059） |  |  |  |  |  |
|  | GG | 165（0.943） | 144 (0.941) |  |  |  |  |  |
| rs7559693  (COL4A3) | GG | 11 (0.063) | 4 (0.026) | 0.223 | 0.023 | 0.241 | 2.651 | 1.232（0.869-1.746） |
|  | GC | 78 (0.446) | 67 (0.438) |  |  |  |  |  |
|  | CC | 86 (0.491) | 82 (0.536) |  |  |  |  |  |
| rs56108400  (AC009779.3) | TT | 20 (0.114) | 16 (0.1051) | 0.848 | 0.909 | 0.540 | 5.940 | 1.107（0.799-1.534） |
|  | TG | 80 (0.457) | 66 (0.431) |  |  |  |  |  |
|  | GG | 75 (0.429) | 71 (0.464) |  |  |  |  |  |
| rs28495773  (PILRB) | TT | 0 (0.000) | 0 (0.010) | 0.726 | 0.708 | 0.782 | 8.602 | 0.876（0.343-2.236） |
|  | TA | 9 (0.052) | 9 (0.059) |  |  |  |  |  |
|  | AA | 165 (0.948) | 144 (0.941) |  |  |  |  |  |
| rs9928736  (BCAR1) | CC | 5（0.029） | 3 (0.020) | 0.076 | 0.705 | 0.210 | 2.310 | 0.751（0.480-1.176） |
|  | CT | 32（0.183） | 41 (0.268) |  |  |  |  |  |
|  | TT | 138（0.789） | 109 (0.712) |  |  |  |  |  |
| rs11777697  (TNFRSF10A) | GG | 19（0.109） | 24 (0.157) | 0.121 | 0.060 | 0.615 | 7.161 | 1.086（0.788-1.495） |
|  | GC | 91（0.520） | 59 (0.386) |  |  |  |  |  |
|  | CC | 65（0.371） | 70 (0.458) |  |  |  |  |  |
| rs4381465  (B3GLCT) | AA | 0 (0.000) | 1 (0.007) | 0.309 | 0.384 | 0.042 | 0.462 | 0.576（0.338-0.985） |
|  | AT | 25 (0.143) | 34 (0.222) |  |  |  |  |  |
|  | TT | 150 (0.857) | 118 (0.771) |  |  |  |  |  |

HWE: Hardy–Weinberg equilibrium; P < 0.05 was considered significant, Bonferroni multiple comparisons.

**Table S5 The genotype frequencies and association analysis of 11 SNPs in AMD cases and controls（71-80 years）**

| SNP  (gene name) | Genotype frequency (%) | | | HWE | | Allele P | Corrected | OR (95%CI) |
| --- | --- | --- | --- | --- | --- | --- | --- | --- |
|  | Genotype | AMD cases | Healthy Controls | AMD | Controls |  | P |  |
| rs760975  (BAIAP2L2) | GG | 6（0.033） | 10（0.042） | 0.980 | 0.133 | 0.583 | 5.357 | 1.106（0.772-1.583） |
|  | GC | 54（0.300） | 60（0.253） |  |  |  |  |  |
|  | CC | 120（0.667） | 167（0.705） |  |  |  |  |  |
| rs11528744  (HTRA1) | CC | 34（0.189） | 48（0.203） | 0.737 | 0.970 | 0.824 | 2.871 | 0.969（0.736-1.277） |
|  | CT | 91（0.506） | 117（0.494） |  |  |  |  |  |
|  | TT | 55（0.306） | 72 (0.304) |  |  |  |  |  |
| rs3761159  (SLC12A5-AS1) | CC | 6（0.033） | 9 (0.038) | 0.282 | 0.448 | 0.959 | 1.320 | 1.009（0.723-1.407） |
|  | CT | 66（0.367） | 84 (0.354) |  |  |  |  |  |
|  | TT | 108（0.600） | 144 (0.608) |  |  |  |  |  |
| rs7212510  (TMEM199) | TT | 15（0.083） | 19 (0.081) | 0.910 | 0.692 | 0.951 | 1.375 | 0.991（0.733-1.340） |
|  | TA | 75（0.417） | 100 (0.426) |  |  |  |  |  |
|  | AA | 90（0.500） | 116 (0.494) |  |  |  |  |  |
| rs6965458  (PILRB) | AA | 2（0.000） | 2（0.000） | 0.002 | 0.019 | 0.866 | 10.439 | 0.943（0.479-1.857） |
|  | AG | 11（0.057） | 17（0.059） |  |  |  |  |  |
|  | GG | 166（0.943） | 218 (0.941) |  |  |  |  |  |
| rs7559693  (COL4A3) | GG | 18 (0.101) | 14 (0.062) | 0.867 | 0.298 | 0.004 | 0.044 | 1.582（1.155-2.167） |
|  | GC | 76 (0.425) | 73 (0.323) |  |  |  |  |  |
|  | CC | 85 (0.475) | 139 (0.615) |  |  |  |  |  |
| rs56108400  (AC009779.3) | TT | 28 (0.156) | 21 (0.089) | 0.714 | 0.361 | 0.042 | 0.462 | 1.345（1.009-1.793） |
|  | TG | 83 (0.461 | 109 (0.460) |  |  |  |  |  |
|  | GG | 69 (0.383) | 107 (0.451) |  |  |  |  |  |
| rs28495773  (PILRB) | TT | 1 (0.006) | 1 (0.004) | 0.104 | 0.246 | 0.894 | 9.834 | 0.952（0.460-1.969） |
|  | TA | 11 (0.062) | 16 (0.068) |  |  |  |  |  |
|  | AA | 166 (0.933) | 218 (0.928) |  |  |  |  |  |
| rs9928736  (BCAR1) | CC | 1（0.006） | 8 (0.034) | 0.387 | 0.320 | 0.038 | 0.418 | 0.647（0.428-0.979） |
|  | CT | 37（0.207） | 59 (0.250) |  |  |  |  |  |
|  | TT | 141（0.788） | 169 (0.716) |  |  |  |  |  |
| rs11777697  (TNFRSF10A) | GG | 17（0.094） | 18 (0.077) | 0.184 | 0.793 | 0.070 | 7.777 | 1.315（0.978-1.768） |
|  | GC | 89（0.494） | 97 (0.413) |  |  |  |  |  |
|  | CC | 74（0.411） | 120 (0.511) |  |  |  |  |  |
| rs4381465  (B3GLCT) | AA | 1 (0.006) | 0 (0.000) | 0.675 | 0.178 | 0.286 | 3.146 | 0.744（0.432-1.283） |
|  | AT | 20 (0.112) | 38 (0.162) |  |  |  |  |  |
|  | TT | 179 (0.883) | 197 (0.838) |  |  |  |  |  |

HWE: Hardy–Weinberg equilibrium; P < 0.05 was considered significant, Bonferroni multiple comparisons.

**Table S6 The genotype frequencies and association analysis of 11 SNPs in AMD cases and controls（>80 years）**

| SNP  (gene name) | Genotype frequency (%) | | | HWE | | Allele P | Corrected | OR (95%CI) |
| --- | --- | --- | --- | --- | --- | --- | --- | --- |
|  | Genotype | AMD cases | Healthy Controls | AMD | Controls |  | P |  |
| rs760975  (BAIAP2L2) | GG | 2（0.056） | 5（0.063） | 0.135 | 0.108 | 0.521 | 5.731 | 0.781（0.367-1.662） |
|  | GC | 7（0.194） | 20（0.250） |  |  |  |  |  |
|  | CC | 27（0.750） | 55（0.688） |  |  |  |  |  |
| rs11528744  (HTRA1) | CC | 9（0.250） | 10（0.125） | 0.335 | 0.786 | 0.096 | 1.056 | 1.615（0.916-2.848） |
|  | CT | 15（0.417） | 35（0.438） |  |  |  |  |  |
|  | TT | 12（0.333） | 35 (0.438) |  |  |  |  |  |
| rs3761159  (SLC12A5-AS1) | CC | 3（0.083） | 7 (0.088) | 0.016 | 0.487 | 0.090 | 0.990 | 0.544（0.267-1.109） |
|  | CT | 6（0.167） | 29 (0.363) |  |  |  |  |  |
|  | TT | 27（0.750） | 44 (0.550) |  |  |  |  |  |
| rs7212510  (TMEM199) | TT | 2（0.056） | 6 (0.075) | 0.664 | 0.358 | 0.666 | 7.326 | 1.151（0.608-2.179） |
|  | TA | 15（0.417） | 26 (0.325) |  |  |  |  |  |
|  | AA | 19（0.528） | 48 (0.600) |  |  |  |  |  |
| rs6965458  (PILRB) | AA | 0（0.000） | 1（0.013） | 0.933 | 0.060 | 0.188 | 2.068 | 0.268（0.033-2.181） |
|  | AG | 1（0.028） | 6（0.075） |  |  |  |  |  |
|  | GG | 35（0.972） | 73 (0.913) |  |  |  |  |  |
| rs7559693  (COL4A3) | GG | 1 (0.028 | 5 (0.063) | 0.096 | 0.463 | 0.871 | 9.581 | 1.052（0.569-1.944） |
|  | GC | 19 (0.528) | 35 (0.438) |  |  |  |  |  |
|  | CC | 16 (0.444) | 40 (0.500) |  |  |  |  |  |
| rs56108400  (AC009779.3) | TT | 2 (0.056) | 4 (0.050) | 0.134 | 0.462 | 0.226 | 2.486 | 1.451（0.792-2.658） |
|  | TG | 20 (0.556) | 33 (0.413) |  |  |  |  |  |
|  | GG | 14 (0.389) | 43 (0.538) |  |  |  |  |  |
| rs28495773  (PILRB) | TT | 0 (0.000) | 1 (0.013) | 0.933 | 0.060 | 0.188 | 2.068 | 0.268（0.033-2.181） |
|  | TA | 1 (0.028) | 6 (0.075) |  |  |  |  |  |
|  | AA | 35 (0.972) | 73 (0.913) |  |  |  |  |  |
| rs9928736  (BCAR1) | CC | 1（0.029） | 1 (0.013) | 0.817 | 0.553 | 0.420 | 4.620 | 1.359（0.644-2.867） |
|  | CT | 11（0.314） | 21 (0.263) |  |  |  |  |  |
|  | TT | 23（0.657） | 58 (0.725) |  |  |  |  |  |
| rs11777697  (TNFRSF10A) | GG | 3（0.083） | 9 (0.113) | 0.777 | 0.911 | 0.699 | 7.689 | 0.888（0.488-1.618） |
|  | GC | 16（0.444） | 35 (0.438) |  |  |  |  |  |
|  | CC | 17（0.472） | 36 (0.450) |  |  |  |  |  |
| rs4381465  (B3GLCT) | AA | 0 (0.000) | 1 (0.013) | 0.933 | 0.804 | 0.020 | 0.220 | 0.127（0.016-0.975） |
|  | AT | 1 (0.028) | 14 (0.175) |  |  |  |  |  |
|  | TT | 35 (0.972) | 65 (0.813) |  |  |  |  |  |

HWE: Hardy–Weinberg equilibrium; P < 0.05 was considered significant, Bonferroni multiple comparisons.
